# Varicella, or Chicken-Pox

**Published:** 1838-08-01

**Authors:** N. Chapman

**Affiliations:** Professor of the Theory and Practice of Physic, in the University of Pennsylvania


### THE MEDICAL EXAMINER.

DEVOTED TO MEDICINE, SURGERY, AND THE COLLATERAL SCIENCES.

EDITED BY J. B. BIDDLE, M. D. AND M. CLYMER, M. D.

No. 16.] PHILADELPHIA, WEDNESDAY, AUGUST 1, 1838. [Vol. I.

### VARICELLA, OR CHICKEN-POX.

*By* N. Chapman, M. D., *Professor of the Theory and Practice of Physic, in the University of Pennsylvania.*

[Reported for this Journal.]

The first of these titles is the diminutive of variola, expressive of a slighter degree of that disease, and the second was probably conferred from the resemblance of the varicellous affection to a similar one in chickens. The poultry of England, according to Jenner, have such an eruption—and he further states, that "in Bengal, they are subject to a disease very like small-pox, which prevails epidemically, and is very fatal. Chickens, there, are inoculated, to preserve their lives." *

It was early remarked, in the history of small-pox, even by Rhazes, that certain exanthematous affections, analogous to it, occasionally appeared, which afforded no protection against that disease. These were subsequently described, about three hundred and fifty years ago, by Vedius, under the title of chrystalli, or chrystalline pock, by reason of the vesicular, rather than the pustular nature of the eruption—and, from that time, a pretty regular account of varicella has been transmitted. By Sydenham it is especially noticed, as a spurious variety of variola, and such seems to have been the view commonly entertained of it. But it is probable that the popular notion in relation to the affection was otherwise, since various vulgar epithets, implying a distinction, were invented in the different countries of Europe, and among these, the English terms, chicken-pock, swine-pock, and hives. It is, indeed, acknowledged by Fuller, in his work, printed in 1730, describing the eruption by this title, that he borrowed it "from the nurses." Moreton, who lived contemporaneously, or nearly so, with him, adopted the same designation, and henceforward, it is every where to be met with.

The first scientific examination of the subject, however, was by Heberden, who, in a paper published in 1767,† contended with great force of argument, that varicella depends on a contagion, specifically different from that of variola, and clearly pointed out the difference in the two diseases, which came to be the universally received doctrine, till, recently, its validity was questioned.

An attack of varicella is marked by few peculiarities. Commencing as most fevers do, and continuing in various degrees of severity, till the expiration of the second or third day, the eruption begins to be disclosed. Yet, for the most part, the fever is moderate, and attracts little or no attention. The eruption is, usually, vesicular, appearing on the face, next on the breast and back, and, ultimately, on the extremities, occasionally preceded by a rash, which is attended by some itching or tingling. It comes out, in successive crops, so that some of the vesicles are matured, some shrivelled away, and some just emerging, thus presenting every stage of progression, and this consecutive train may be maintained for several days. "They are about the size of a split pea, perfectly transparent, covered simply by the cuticle, and, when the eruption is copious, the body has the appearance of having been exposed to a shower of boiling water, each drop of which had occasioned a minute blister." There are, however, varieties in the configuration of the vesicles, which were early recognised, and discriminated by the popular names of chicken-pox, swine-pox, and hives, and since, more classically entitled by Willan, lenticular, conoidal, and globate varicella, according to the respective forms.

To Bateman, and other writers of easy access, by whom all the peculiarities of these modifications of the disease, have been minutely indicated, I must refer for such information. It may be succinctly stated, that the eruption, thus undoubtedly diversified as to shape, is sometimes, also, characterized by an earlier or later appearance—by its being oftener purely vesicular throughout its existence, though in other instances more pustular, as well in figure as the contained fluid,—that, without any secondary fever, it begins to dry and scab, in the order of its successive occurrence,—the whole falling off by desquamation, in four, six, or eight days, seldom leaving any pits, or other vestiges.

But though such are the ordinary aspects and course of varicella, it occasionally exhibits itself very differently. The fever is infinitely more vehement, the local affections of the stomach, lungs, and brain, correspondently heightened, and the case as well in the character and extent of the eruption, as in other features, approaching very closely to small-pox. An account of an epidemic varicella of this exasperated nature, is given by Sims, as having prevailed in London, some fifty years ago, and sporadic cases of a similar kind are familiar to us in this city.

That varicella belongs to the class of diseases, proceeding from a specific infection, is not disputed. But, whether this be distinct, or the same as that of variola, is a point still under discussion—and the progress of opinion, in relation to it, has been already adequately traced. It is by Professor Thompson, that the views as settled for a time by Heberden, have been endeavoured to be reversed or overthrown, to revive, again, the ancient notion, that varicella is really modified small-pox. To this conclusion, he was led by a very attentive observation of the phenomena of the epidemic variolous disease, at Edinburgh in 1818, and confirmed by a very elaborate research into the subject. Expressed in a few words, these are his arguments:

1. That varicella and variola may each communicate the other by a subjection to their respective contagions, by inoculation, or through the atmosphere.

* Barron's Life of Jenner, p. 237.

† Med. Trans. Coll. Phys., London, vol. I.

2. That neither disease ever prevails epidemically, without the other being, at the same time, observable.

3. That varicella never shows itself, except in persons, who previously have had variola or vaccinia, and, hence, that it is the product of the variolous virus operating on a system thus modified.

It must be confessed, that these positions are sustained by an immense mass of facts, and by a chain of very cogent and plausible reasoning. Yet few converts have been made to them, and the weight of authority still decidedly preponderates in favour of what was antecedently considered as the established doctrine.

Let us next see how this side of the question is supported, and, to attain greater perspicuity, I shall take up the adverse positions just mentioned, in the order in which they are presented.

1. It is replied, that it is exceedingly difficult, while the two diseases are simultaneously prevailing, to assign the origin of a case of the one, to the infection from the other, as the contagion of both is diffused at the same moment, through the atmosphere. Nor is it true, that the two diseases can be mutually produced by inoculation. The virus of variola invariably causes small-pox, and it is altogether doubtful, whether varicella be capable of propagation in this mode. Experiments, which go to prove it, are contradicted by a host of an opposite description, and it is not unreasonable to presume, that, in the few successful trials alleged, the matter procured was variolous. On this point, the language of the late Mr. Bryce, of Edinburgh, a most respectable authority, is very decisive. It is stated by him, "that he has taken lymph from the vesicles of true varicella, with the greatest care, at all periods of the disease, and all seasons of the year—that he himself has inoculated, and seen others inoculate with it, children, who had never undergone either small or cow-pox, to the number of thirteen, yet, in none of these, was this disease, or any thing like small-pox ever produced." *

2. That the allegation of the uniform simultaneous prevalence of varicella and variola, is not borne out by the history of the diseases. The fact is, that though they do occasionally co-exist, the reverse oftener happens. Much proof might be adduced of their separate and independent prevalence. Enough, however, may it be to state, that we are assured, that, from 1809 to 1823, varicella was annually observed at Copenhagen, without variola, and that it is well known, for quite as long a period, the same happened in this city.

3. That it is erroneous to suppose, that varicella cannot exist in a system which has not undergone the variolous or vaccine infection. Common experience denies it. Examples of the kind were very familiar prior to the introduction of vaccination, when the unprotected system was more frequently to be met with, and are still sometimes witnessed.

To the preceding objections to the hypothesis I am combating, it may be added, as calculated further to its invalidation, that vaccination practised subsequently to varicella, pursues its ordinary course, which it does not after small-pox, and that varicella undergoes no change in a system modified by the influence of vaccinia or variola. Finally, varicella, with few exceptions, is distinct from small-pox, in the character of the eruption, the prelusive and attendant symptoms, and in every other material respect.

Enough has been said of the discriminative signs of this disease—and its prognosis, as well as anatomical characters, may, hence, be so readily deduced, as not to require to be indicated. Of its pathology, I have only to add to the remarks already incidentally delivered concerning it, that varicella is, unquestionably, one of those diseases in which the system loses its susceptibility to a second attack, or, if such occasionally happens, it must be deemed an anomalous deviation from the tenor of its character. It occurs almost exclusively in infancy and childhood, so strictly so, that some have denied its existence at all, in adult or more advanced life. But this, perhaps, is going too far, as Willan, Gregory, and other of our best authorities, have observed the contrary, though the instances on record, well authenticated, are extremely few, and the question may be considered as not satisfactorily determined. Existing sometimes sporadically or sparsely, it much more commonly prevails epidemically, under which circumstances, it makes a wide sweep, among children, running through whole families, or the larger collection of them, in schools. This embraces all which I deem of importance in relation to the history of varicella. As to the treatment, little is usually required to be done—and when a case assumes a more serious aspect, a course similar to that in mild small-pox may be adopted.

* Thompson on Varioloid Disease, p. 74.

